# Sequencing, *De Novo* Assembly, and Annotation of the Complete Genome of a New Thraustochytrid Species, Strain CCAP_4062/3

**DOI:** 10.1128/genomeA.01335-17

**Published:** 2018-03-15

**Authors:** Khawla Seddiki, François Godart, Riccardo Aiese Cigliano, Walter Sanseverino, Mohamed Barakat, Philippe Ortet, Fabrice Rébeillé, Eric Maréchal, Olivier Cagnac, Alberto Amato

**Affiliations:** a Laboratoire de Physiologie Cellulaire et Végétale, UMR 5168 CNRS, CEA, INRA, Université Grenoble Alpes, Biosciences and Biotechnology Institute of Grenoble, CEA-Grenoble, Grenoble, France; b Fermentalg, Libourne, France; c Sequentia Biotech SL, Barcelona, Spain; d Aix Marseille Université, CEA, CNRS, UMR7265, LEMIRE, Laboratoire d’Ecologie Microbienne de la Rhizosphère et Environnement Extrêmes, ECCOREV FR3098, St. Paul Les Durance, France

## Abstract

Thraustochytrids are ecologically and biotechnologically relevant marine species. We report here the *de novo* assembly and annotation of the whole-genome sequence of a new thraustochytrid strain, CCAP_4062/3. The genome size was estimated at 38.7 Mb with 11,853 predicted coding sequences, and the GC content was scored at 57%.

## GENOME ANNOUNCEMENT

Thraustochytrids are marine unicellular heterokont eukaryotes. Although their distribution in aquatic ecosystems can be broad, up to a depth of 4,000 m, they colonize mainly mangrove habitats (1). They are characterized by (i) monocentric thallus, (ii) ectoplasmic net systems, (iii) scaly cell walls, and (iv) intricate life cycles (2). The ability of some species to produce long-chain polyunsaturated fatty acids has attracted industrial interest. Four thraustochytrid strains have been sequenced to date (3, 4). Here, we present the genome of a new thraustochytrid species, which is highly similar to Aurantiochytrium limacinum/mangrovei and Schizochytrium aggregatum. The taxonomic position of this new species will be determined later, after more thorough analyses.

The pollen grain bait isolation method (5) was used to obtain monospecific axenic cultures from coastal seawater samples gathered in Mayotte (Indian Ocean, 12°48′51.8″S, 45°14′21.7″E). Once the culture was proved to be monospecific and axenic, it was deposited at the Culture Collection of Algae and Protozoa (CCAP) under the accession number CCAP_4062/3.

Cells were propagated in M1 medium (6) at 30°C under agitation (140 rpm). Genomic DNA was extracted and purified using a Quick-DNA Miniprep Plus kit (Zymo Research) according to the manufacturer’s instructions. Quality and concentration were estimated using the Quant-iT PicoGreen dsDNA assay kit. Shotgun sequencing libraries were prepared and sequenced as paired-end reads (100 bp) on an Illumina HiSeq platform (Illumina, Inc.). The quality of the raw reads was assessed with FastQC (http://www.bioinformatics.babraham.ac.uk/projects/fastqc).

About 137,997,490 paired-end reads were *de novo* assembled using SPAdes version 3.10.1 (7) with a multi-*k*-mer approach (*k* = 27, 31, 35, 41, 45, 49, and 53). Contigs were scaffolded with Satsuma using the genome of Schizochytrium sp. CCTCC M209059 (GenBank accession number JTFK00000000) as the reference. The final assembly consisted of 4,503 contigs ≥300 bp assembled in 2,232 scaffolds. The *N*_50_ value was 236,568 bp, and the overall GC content was 57%. AUGUSTUS version 3.3 was used for gene prediction (8). A total of 11,853 coding sequences were recorded. BUSCO (9) analysis showed that 10% of the eukaryotic conserved gene core is not represented and that 85.1% is represented by complete single-copy genes in our genome.

Functional annotation was carried out using AHRD (https://github.com/groupschoof/AHRD) after a BLAST search against the UniProt Eukaryote and Protist TrEMBL protein database was performed. A description was associated with 7,861 sequences (66%), while a gene ontology (GO) annotation was assigned to 7,269 transcripts (61%). Analysis with barrnap software (https://github.com/tseemann/barrnap) identified three copies of the 5.8S gene, eight partial copies of the 28S gene, and seven partial copies of the 18S gene. The 18S and 28S sequences were finally reconstructed by PCR amplification and direct Sanger sequencing.

The taxonomy and systematics of this strain are under study. This genome is considered of high interest, because metabolic pathways potentially suitable for the bioproduction of different high-added-value molecules can be identified. Other Labyrinthulomycetes genomes have been sequenced, and comparative analyses of closely related taxa can lead to a better understanding of the evolutionary success of thraustochytrids, particularly their ability to produce considerable amounts of lipids.

### Accession number(s).

This whole-genome shotgun project has been deposited at DDBJ/EMBL/GenBank under BioProject PRJNA396017. The version described in this paper is the first version. The 18S, 5.8S, and 28S rRNAs have been deposited at GenBank under the accession numbers MF766427, MF766428, and MF766429, respectively.
